# *Cyclocarya paliurus* extract attenuates hepatic lipid deposition in HepG2 cells by the lipophagy pathway

**DOI:** 10.1080/13880209.2020.1803365

**Published:** 2020-09-02

**Authors:** Wanwei Yang, Cuihua Jiang, Zhiguo Wang, Jian Zhang, Xiaodong Mao, Guofang Chen, Xiaoming Yao, Chao Liu

**Affiliations:** a Clinical Laboratory, Affiliated Hospital of Integrated Traditional Chinese and Western Medicine, Nanjing University of Chinese Medicine, Jiangsu Province Academy of Traditional Chinese Medicine, Nanjing, Jiangsu, PR China; b Laboratory of Translational Medicine, Affiliated Hospital of Integrated Traditional Chinese and Western Medicine, Nanjing University of Chinese Medicine, Jiangsu Province Academy of Traditional Chinese Medicine, Nanjing, Jiangsu, PR China; c Research Center of Endocrine and Metabolic Diseases, Affiliated Hospital of Integrated Traditional Chinese and Western Medicine, Nanjing University of Chinese Medicine, Jiangsu Province Academy of Traditional Chinese Medicine, Nanjing, Jiangsu, PR China

**Keywords:** Non-alcoholic fatty liver disease, lipid metabolism, autophagy

## Abstract

**Context:**

*Cyclocarya paliurus* (CP) (Batal.) Iljinsk (Cyclocaryaceae), a plant native to China, is the sole species in the genus *Cyclocarya*. Its leaves have been widely used as a remedy for hyperlipidaemia in traditional folk medicine. However, the mechanism underlying CP-induced lipolysis, especially in the liver, has not been entirely elucidated.

**Objective:**

This study investigates the effect of CP ethanol extract (CPE) on hepatic steatosis and the underlying molecular mechanisms involved.

**Materials and methods:**

The effect of CPE at concentrations of 0, 6.25, 12.5, 25, 50, and 100 μg/mL on the viability of HepG2 cells was examined using the cell counting kit-8 (CCK-8) assay after incubation for 24 h. CPE-induced changes in intracellular lipid content were assessed by measuring the absorbance of oil red O staining at 520 nm, and the possible underlying mechanisms were further studied using quantitative reverse transcription polymerase chain reaction (RT-qPCR) analysis, western blotting, immunofluorescence studies and transmission electron microscopy.

**Results:**

The half-maximal inhibitory concentration (IC_50_) of CPE in HepG2 cells was 97.27 μg/mL. Treatment with 50 μg/mL CPE increased lipid clearance, which was associated with increased autophagy in HepG2 cells. CPE-induced autophagy involved downregulation of phosphorylation level of mammalian target of rapamycin (0.87 ± 0.08 *vs.* 1.31 ± 0.10). Fluorescent double staining and electron microscopy images showed lipid deposits within autolysosomes, thereby confirming the abovementioned findings.

**Discussion and conclusions:**

CPE can induce hepatic fat clearance through the autophagy-lysosome pathway known as lipophagy. CPE has potential as a functional food.

## Introduction

Non-alcoholic fatty liver disease (NAFLD) has become common in conjunction with the increasing prevalence of metabolic syndrome, which encompasses a wide spectrum of conditions, from fatty liver to non-alcoholic steatohepatitis (NASH), fibrosis, and cirrhosis (Buzzetti et al. 2016; Cobbina and Akhlaghi 2017). Since there are no medical treatments for NASH, it has become the third leading indication for liver transplantation in the United States and is on a trajectory to become the most common indication (Charlton et al. 2011). Hence, early improvements in hepatic fat deposition, preventing the progression of fatty liver into NASH, play a pivotal role in the treatment of NAFLD.

*Cyclocarya paliurus* (Batal.) Iljinsk (Cyclocaryaceae; CP), commonly known as ‘sweet tea tree’, is the sole species in the genus *Cyclocarya*. This plant only grows in the highland of southern China (Shu et al. 1995). Numerous studies have shown that CP has multiple bioactivities and could be beneficial in the treatment of diabetes and hyperlipidaemia (Wang et al. 2013; Lin et al. 2016; Li et al. 2018). We previously reported that an ethanol extract of CP (CPE) could prevent high-fat diet-induced hyperlipidaemia and obesity in Sprague-Dawley rats; CPE had an especially positive effect on alleviating fat accumulation in the liver (Yao et al. 2015). However, the mechanism of CPE-induced lipid breakdown in the liver is not yet completely understood.

The term autophagy was introduced by Deter et al. (1967) over 50 years ago to define the process of vacuolization for the transport of intracellular material to lysosomes for degradation. Singh et al. (2009) were the first to convincingly correlate autophagy with lipid metabolism. The present study focussed on an alternative pathway of lipid breakdown called lipophagy, which delivers lipid droplets (LDs) to lysosomes for the degradation of LDs. The lipophagic process involves sequestration of LDs in double-membrane autophagosomes, followed by fusion with lysosomes to form autolysosomes and subsequent degradation of the LDs by lipases located within the autolysosomes (Yang et al. 2010; Dong and Czaja 2011). We hypothesized that lipophagy is associated with CPE-induced hepatic fat clearance *in vitro* in order to explain the antisteatotic action of this widely consumed plant in China.

## Materials and methods

### Plant materials and preparation

CP leaves were obtained from cultivated plants in Nanjing Forestry University on October 11, 2017. The samples were identified by Professor Min-jian Qin from China Pharmaceutical University (Nanjing, China), and a voucher specimen (No. L20100033) was stored in the university’s Department of Natural Medicinal Chemistry. CPE was previously analysed by high-performance liquid chromatography to identify its main compounds, which are triterpenoids and flavonoids (Ma et al. 2015). The leaves were air-dried, powdered (2.5 kg) and extracted three times with 20 L of 80% ethanol for 2 h per extraction. The combined extracts were filtered and concentrated under reduced pressure to obtain the crude extract (455 g), which was stored at 4 °C. Before use, the CPE was dissolved in distilled water.

### Chemicals

Cell culture media and serum were purchased from Gibco (Grand Island, NY, USA). Cell counting kit-8 (CCK-8) was obtained from Dojindo (Tokyo, Japan). Oleic acid (OA), palmitic acid (PA) and rapamycin were purchased from Sigma-Aldrich (St. Louis, MO, USA). Western blot antibodies were from Cell Signalling Technology (Beverly, MA, USA). The plasmid expressing red fluorescent protein-tagged LC3 (RFP-LC3) was purchased from Hanbio (Shanghai, China). Lipofectamine 3000 Transfection Reagent and Bodipy 493/503 were obtained from Thermo Fisher Scientific (Waltham, MA, USA). Lyso-tracker red was purchased from Beyotime (Shanghai, China). All other reagents and solvents were of analytical grade, and all aqueous solutions were prepared with double-distilled water.

### Cell culture

The HepG2 cell line was obtained from the Chinese Academy of Sciences (Shanghai, China) and maintained at 37 °C in Minimum Essential Medium (MEM) supplemented with 10% foetal bovine serum. To establish a model of fatty liver cells, a lipid mixture was prepared containing 0.5 mM of OA and PA in a 2:1 ratio diluted in MEM containing 2% (w/v) bovine serum albumin.

### Cell viability assay

The HepG2 cells were seeded overnight in 96-well plates at a density of 2 × 10^4^ cells per well and subsequently treated with the lipid mixture for 16 h. CCK-8 diluted 1:10 in phosphate-buffered saline (PBS) was added to each well and incubated for 1 h, followed by careful removal of the CCK-8 solution. Finally, the absorbance of each well was measured at 450 nm with a Synergy H1 Hybrid Multi-Mode Microplate Reader (BioTek, Winooski, VT, USA). Cell viability was expressed relative to the untreated cell viability in the control group.

### Intracellular fat measurement in HepG2 cells

The cells were subjected to lipid mixture treatment for 16 h, followed by CPE (50 μg/mL) treatment for 24 h. Thereafter, the cells were washed once with PBS and fixed with 10% formaldehyde for 30 min. Then, the cells were dried and stained with oil red O (ORO) solution (0.5 g of ORO powder dissolved in 60% isopropanol) for 10 min at 25 °C. The cells were washed thrice in water and images were acquired on an Olympus IX71 microscope. To quantify the ORO content, isopropanol was added to each sample, followed by shaking at 25 °C for 10 min. The absorbance was then measured at 520 nm on a spectrophotometer (BioTek).

### RNA isolation and quantitative reverse transcription polymerase chain reaction (RT-qPCR)

Total RNA extraction from the HepG2 cells was performed with an RNeasy Mini Kit (Qiagen, Dusseldorf, Germany), and the RNA concentrations were assessed by absorbance at 260 nm with a Synergy H1 Hybrid Multi-Mode Microplate Reader. cDNA was synthesized from the total RNA with the ReverTra Ace qPCR RT Kit (TOYOBO, Osaka, Japan). qPCR was performed using the SYBR Green PCR Kit (TOYOBO) according to the manufacturer’s instructions. GAPDH levels were used for normalization, and fold changes were calculated using 2^−△△Ct^. The primer sequences are available upon request.

### Immunofluorescence studies

For autophagic analysis, the RFP-LC3 plasmid was transfected into HepG2 cells with Lipofectamine 3000 transfection reagent according to standard protocols. For Lyso-tracker red and Bodipy 493/503 staining, the cells were grown on glass coverslips and treated with the lipid mixture for 16 h, followed by CPE treatment for 24 h. Thereafter, the cells were incubated with Lyso-tracker red (50 nmol/L) for 30 min at 37 °C. After PBS washes, the cells were incubated with Bodipy 493/503 at 1 μg/mL for 5 min at 37 °C, followed by three PBS washes. The coverslips were observed under an Olympus FV10i confocal microscope (Japan). Image colocalization was performed using Image J (NIH, Bethesda, MD).

### Western blotting

Protein was extracted from cells with lysis buffer containing Pierce protease inhibitor tablets (Thermo Fisher Scientific). Protein concentration was then determined using a bicinchoninic acid assay (Biotime, Shanghai, China). Equivalent amounts of protein were resolved by sodium dodecyl sulfate-polyacrylamide gel electrophoresis (SDS-PAGE, 10% gel; Biotime) and transferred to PVDF membranes using a BioRad blotting system. After blocking with 5% (w/v) non-fat dried milk, the membranes were incubated with monoclonal antibodies (1:1000–1:5000) at 4 °C overnight. Then, the membranes were washed three times with Tris-buffered saline containing 0.1% Tween 20 and incubated with horseradish peroxidase-conjugated secondary antibodies (1:10000; Sigma-Aldrich) for 1 h. Bands were visualized using an enhanced chemiluminescence system (Millipore, Billerica, MA, USA). Densitometry analysis was performed using Quantity One software (BioRad, Hercules, CA, USA).

### Transmission electron microscopy (TEM)

HepG2 cells were seeded in a 10 cm dish and treated with the lipid mixture for 16 h, followed by CPE (50 μg/mL) treatment for 24 h. The cells were fixed with 2.5% glutaraldehyde and then treated with 1% osmium tetroxide buffer. Thereafter, the cells were dehydrated with various concentrations of ethanol, transitioned into 100% acetone, and embedded in EMBed-812 resin. The cells were sequentially stained with 2% uranyl acetate and Reynold’s lead citrate after ultramicrotome sectioning (Leica UC7, Germany). TEM images were acquired with a transmission electron microscope (Hitachi, HT7700, Tokyo, Japan).

### Statistical analysis

Cell culture experiments were performed in duplicate and repeated at least three times by using matched controls. Results are expressed as mean ± SD values. Statistical analyses were performed using GraphPad Prism 5.0 (GraphPad Software, San Diego, CA, USA). Statistically significant differences (*p* < 0.05) were assessed using paired *t*-tests or one-way analysis of variance followed by Newman–Keuls *post hoc* tests.

## Results

### Effect of CPE on cell viability of HepG2 cells

**Figure 1.**
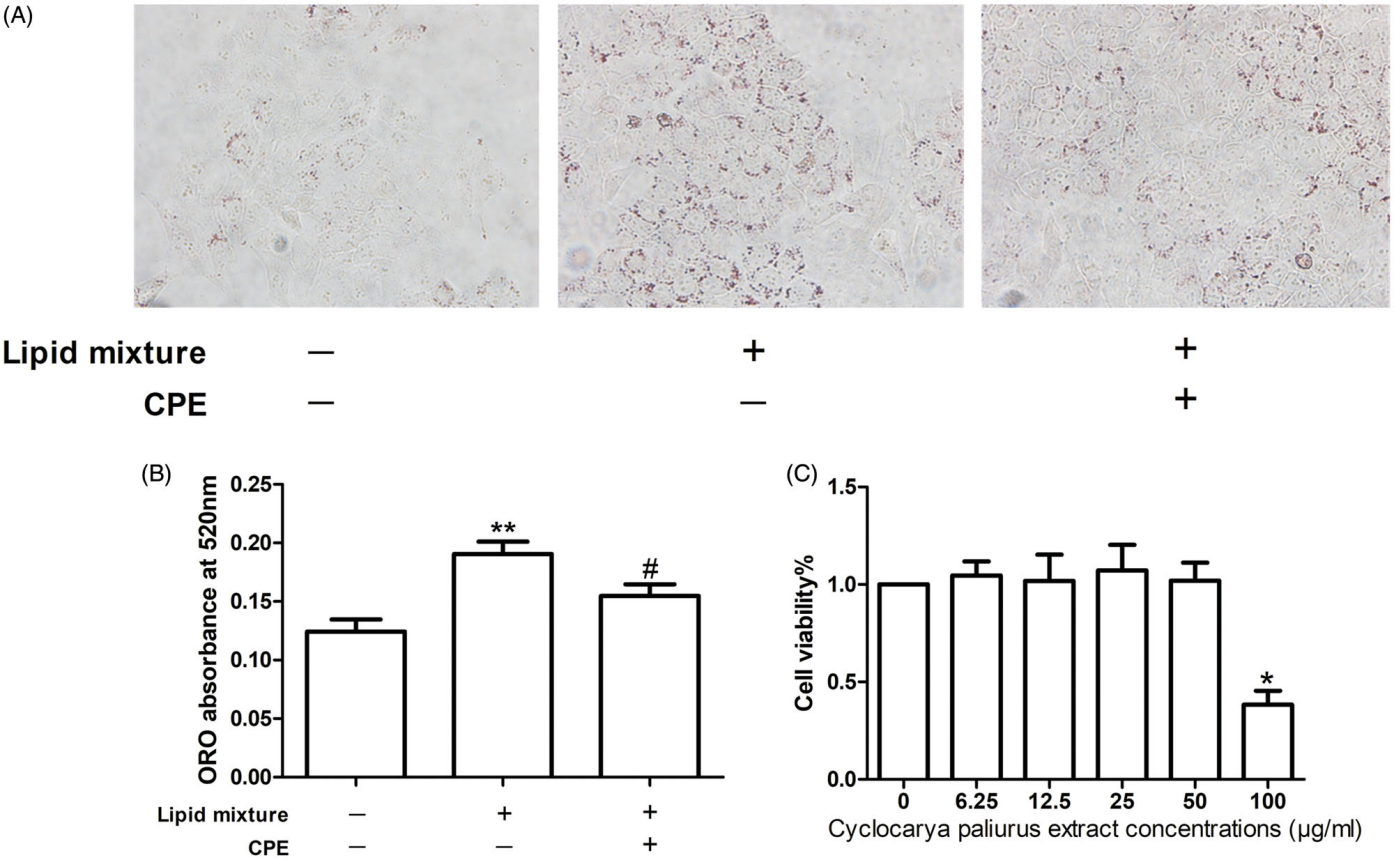
The CPE (50 μg/mL) treatment increased in lipid clearance and had no effect on cell viability of HepG2 cells. (A) Representative image of oil red O (ORO)-stained HepG2 cells, which were treated with 0.5 mM OA and PA combination in a 2:1 ratio for 16 h, followed by 24 h post-treatment with 50 μg/mL of CPE, compared to lipid mixture-treated cells and control cells (no lipid was added) (*n* = 3). (B) Changes in intracellular lipid content were assessed by measuring ORO absorbance at 520 nm. **p < 0.01 vs. no lipid treated cells, #*p* < 0.05 vs. lipid mixture-treated cells. (C) Cell viability was determined by the CCK-8 assay. Values are expressed as percentage of the control. Data represent mean ± SDs of three independent experiments. *p < 0.05 vs. every other group.

The viability of HepG2 cells treated with CPE at concentrations of 0, 6.25, 12.5, 25, 50, and 100 μg/mL was evaluated using the CCK-8 assay. CPE treatment did not result in any apparent reduction in viability at concentrations ≤50 μg/mL. Cell viability of the group of cells treated with 100 μg/mL CPE was significantly decreased compared to that of other groups; the IC_50_ value was 97.27 μM (Figure 1(C)). Thus, we used a CPE concentration of 50 μg/mL in subsequent experiments.

### CPE (50 μg/mL) treatment induces lipid clearance in HepG2 cells

Increased hepatocellular lipid deposition after lipid mixture treatment for 16 h appeared as ORO-stained red particles, while CPE treatment led to lipid breakdown (Figure 1(A)). These findings were also observed in the ORO absorbance assay at 520 nm, which measures intracellular lipid content (Figure 1(B)).

### CPE-induced lipid reduction is associated with increased autophagy in HepG2 cells

**Figure 2.**
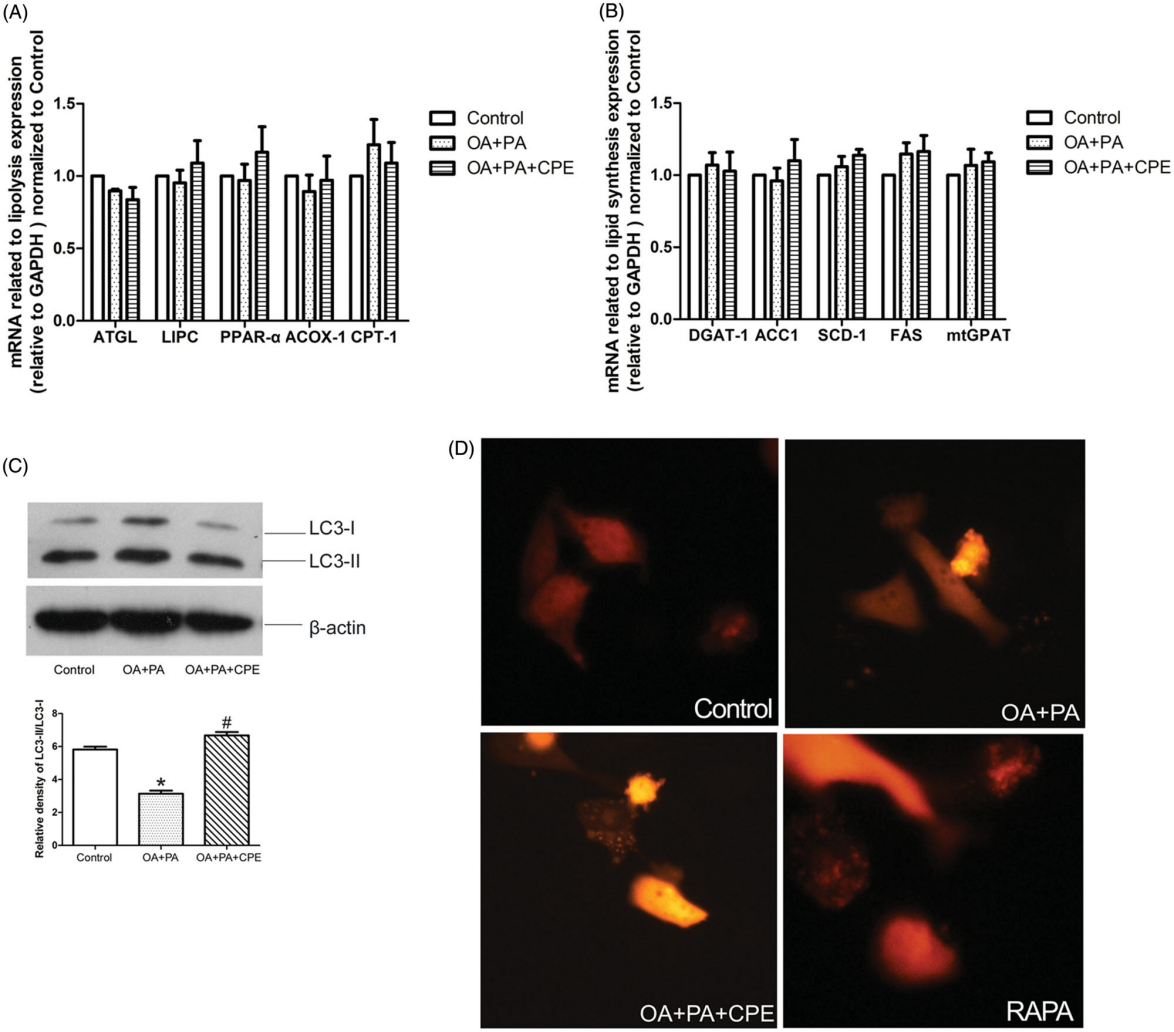
CPE-induced lipid clearance was temporally coupled with an increase in LC3 expression without affecting the classical lipolysis and lipid synthesis pathway. (A, B) The data showed mRNA levels of genes regulating lipolysis and lipid synthesis in HepG2 cells, which were treated with 0.5 mM of OA and PA mixture for 16 h, followed by 24 h post-treatment with 50 μg/mL of CPE, compared to OA + PA mixture-treated cells and no lipid-treated cells (control group). Values are presented as means ± SDs (*n* = 6). (C) Representative immunoblotting and densitometric analysis showing LC3-II accumulation in HepG2 cells treated with 50 μg/mL of CPE for 24 h vs. non-treated cells and OA and PA mixture treated cells. *p < 0.05 vs. control group, #*p* < 0.05 vs. OA and PA mixture treated cells (*n* = 3). (D) Representative image of autophagosomes in RFP-tagged LC3 plasmid-transfected HepG2 cells treated with 0.5 mM of OA and PA mixture for 16 h, followed by 24 h of 50 μg/mL of CPE treatment or 1 μM of rapamycin vs. OA and PA mixture-treated cells and control group (*n* = 3).

To investigate the mechanism of CPE-induced lipid turnover, we treated HepG2 cells with the lipid mixture for 16 h, followed by treatment with 50 μg/mL CPE, and then examined the mRNA expression related to lipolysis and lipid synthesis. There were no significant differences in adipose triglyceride lipase, hepatic lipase (LIPC), peroxisome proliferator-activated receptor-α (PPARα), peroxisomal acyl-coenzyme A oxidase 1 (ACOX-1), and carnitine palmitoyltransferase-1 (CPT-1) levels after CPE treatment (Figure 2(A)). There were also no differences in triglyceride packing-related genes, such as diacylglycerol acyltransferase 1 (DGAT1), acetyl-CoA carboxylase 1 (ACC1), stearoyl-CoA desaturase 1 (SCD-1), fatty acid synthase, and mitochondrial glycerol-3-phosphate acyltransferase (mtGPAT) (Figure 2(B)). These results indicate that CPE probably does not affect the routine lipolysis pathway or triglyceride synthesis in HepG2 cells. To further understand the role of autophagy in CPE-induced lipid clearance, we examined autophagosome formation by detecting LC3 expression. CPE-induced lipid turnover was temporally coupled with an increase in LC3II/LC3I expression, indicating increased autophagy (Figure 2(C)). Autophagosome formation was also shown by punctate fluorescence emission of LC3-II after RFP-LC3 plasmid transfection (Figure 2(D)).

### CPE induces autophagy by suppressing the mTOR signalling pathway in hepatic cells

**Figure 3.**
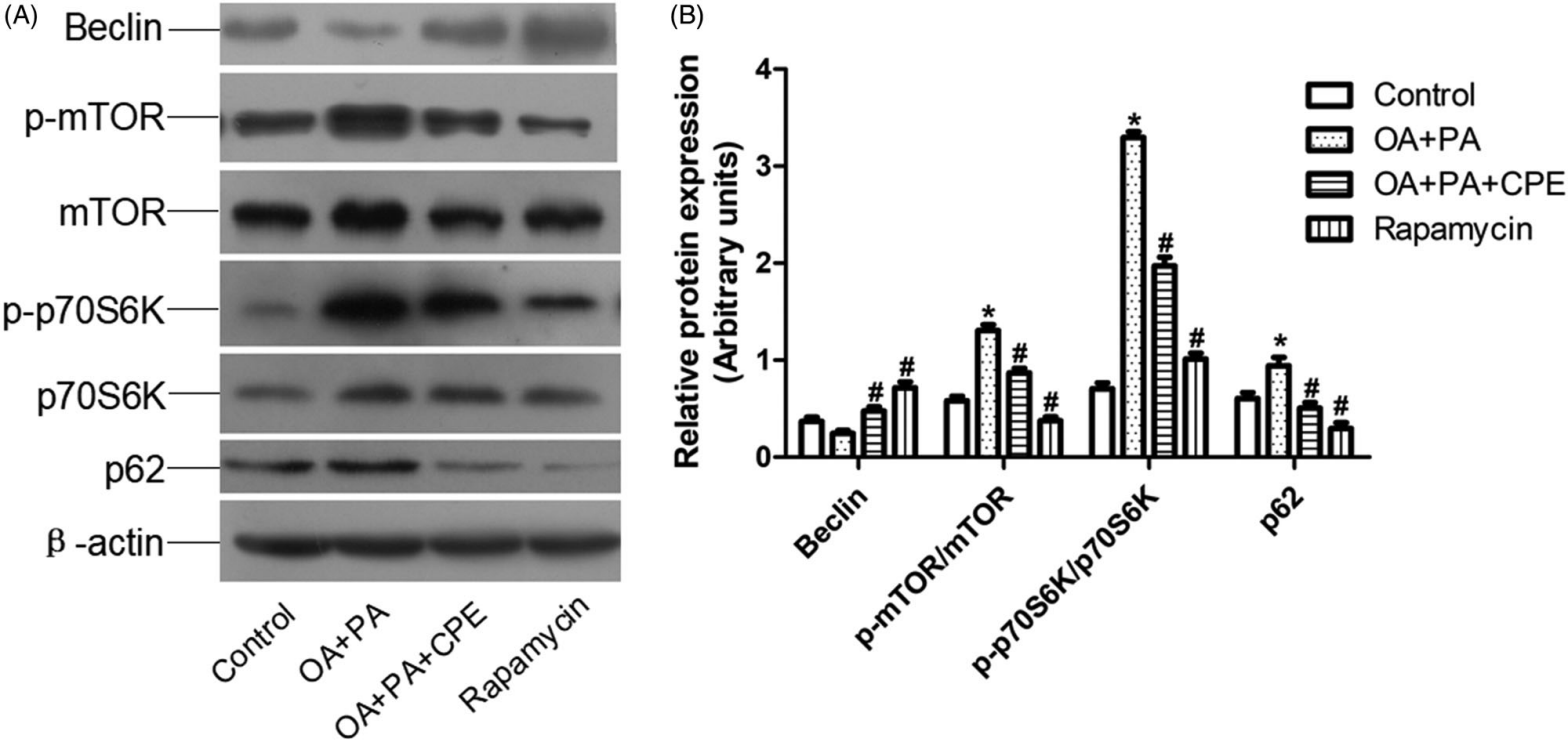
Expression of autophagy-regulated protein in HepG2 cells. (A) Protein expression involved in mTOR signalling pathway of HepG2 cells treated with 50 μg/mL of CPE or 1 μM of rapamycin for 24 h, after 0.5 mM of OA and PA mixture treatment for 16 h vs. OA + PA mixture treated cells and no lipid-treated cells (*n* = 4). (B) Densitometric analyses of the protein expressions normalized to β-actin. The statistical analyses were performed using one-way analysis of variance followed by Newman–Keuls post-hoc tests. Rapamycin was used as an autophagy inducer. *p < 0.05 vs. no lipid-treated cells, #*p* < 0.05 vs. lipid mixture treated cells. The data are presented as means ± SDs (*n* = 4).

Downregulation of mTOR phosphorylation (p-mTOR) and increased expression of Beclin, a proautophagic protein, were associated with CPE-induced autophagy in HepG2 cells after CPE treatment. Additionally, the CPE-treated HepG2 cells exhibited reduced levels of p-p70S6K (Figure 3). Since it is possible that either induction of autophagy or inhibition of autophagosome clearance could account for the increase in LC3-II levels, we also assessed the protein levels of p62 in the cells after CPE treatment. As shown in Figure 3, p62 expression was reduced in the CPE-treated cells, indicating increased autophagic flux. Taken together, these findings demonstrate that the lipophagic pathway is involved in the clearance of intracellular lipid by CPE.

### Autophagy-lysosomal pathway is involved in the reduction in intracellular lipids by CPE

**Figure 4.**
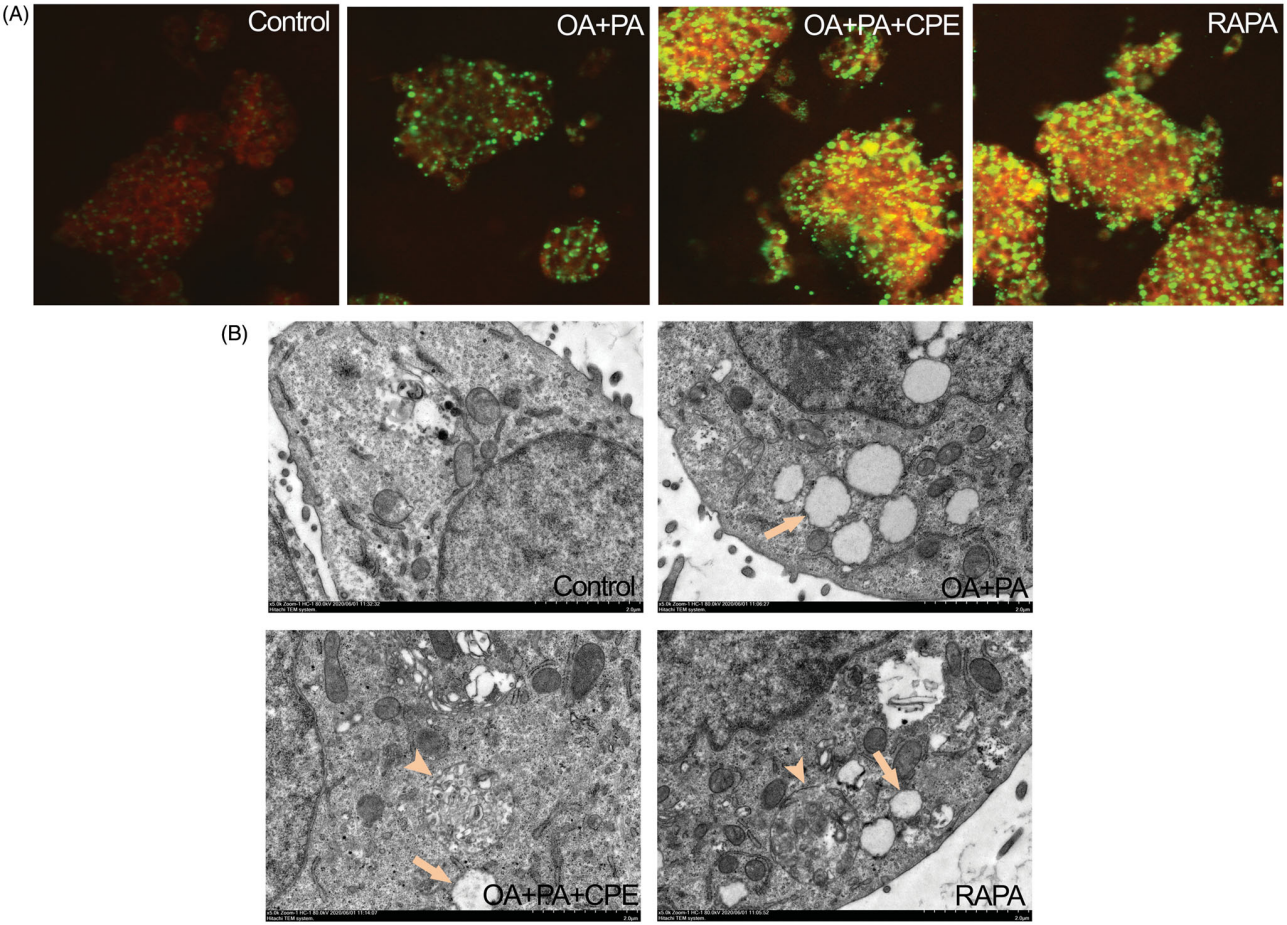
Role of autolysosomes in CPE-mediated clearance of intracellular lipid in HepG2 cells. (A) HepG2 cells treated with 50 μg/mL of CPE or 1 μM of rapamycin (RAPA) for 24 h after 0.5 mM of OA + PA mixture treatment for 16 h, followed by double labelling with Lyso-tracker red (red) and Bodipy493/503 (green) showed increased colocalization of autolysosomes and cellular lipids (yellow dots) vs. OA and PA treated cells and no lipid-treated cells. (B) Electron micrograph images of HepG2 cells treated with 50 μg/mL of CPE or 1 μM of RAPA for 24 h after 0.5 mM of OA + PA mixture treatment for 16 h showed autolysosomes filled with lipids vs. OA and PA treated cells and no lipid-treated cells. Arrow indicated the lipid droplets and autolysosome structure was denoted with arrow heads. RAPA was used as a positive control.

Lysosomal activity was assessed using Lyso-tracker red, a lysosomotropic acidic agent. Confocal microscopy demonstrated increased colocalization of Bodipy 493/503-stained LDs (green) with lysosomes (red) in the CPE-treated cells, shown as yellow dots, since autophagy-induced degradation of lipids occurred in the lysosomes (Figure 4(A)). Consistent with these fluorescence staining results, TEM images further confirmed these findings by showing autolysosomes structure (arrowhead) in the cells treated with 50 μg/mL CPE after treatment with 0.5 mM of the lipid mixture (Figure 4(B)).

## Discussion

In this study, we showed that CPE could effectively decrease intracellular lipids and increase autophagic flux in human hepatic cells. Immunofluorescence studies demonstrated that the autophagy-lysosomal pathway is involved in the reduction in intracellular fat. These results were further corroborated by electron micrographs showing lipids colocalized within autophagosomal and lysosomal compartments, indicating the ingestion of cytosolic lipids by autophagosomes and their subsequent delivery to lysosomes.

When eukaryotic cells are deprived of amino acids, they activate autophagy, which delivers intracellular proteins and organelles sequestered in double-membrane vesicles (autophagosomes) to lysosomes for degradation to use as an energy source (Levine and Klionsky 2004). The autophagy pathway is mediated by a group of proteins, the ATG proteins, the Beclin 1/VPS34 class III phosphatidylinositol kinase (PI3K) complex, and the ATG5/ATG12/ATG16 and Atg8/LC3 protein conjugation complexes (Mizushima and Komatsu 2011; Laplante and Sabatini 2012). As a nutrient-sensing kinase, the target of rapamycin complex 1 (mTORC1) is a central regulator of autophagy. In our study, CPE relieved this inhibitory phosphorylation to initiate the activation of autophagy, which might, in turn, activate the Beclin1-class III phosphatidylinositol-3-kinase (VPS34 or PI3K3) complex to initiate autophagosome formation. Activity of the downstream target p70S6K was downregulated simultaneously. Thus, CPE modulated hepatocellular autophagy through the mTOR/p70S6K pathway.

Autophagy can regulate the intracellular level of lipids through its involvement in LD removal; this process is also named lipophagy (Singh et al. 2009). Inhibition of autophagy by genetic manipulation or drugs increases intracellular lipid content (Ding et al. 2010; Wu et al. 2010). Conversely, enhancing autophagy by overexpressing Atg7 was able to improve hepatic steatosis in ob/ob mice (Yang et al. 2010). These observations demonstrate that pharmacological autophagy modulation, such as that with CPE treatment, may offer a new strategy for treating hepatic lipid deposition, taking into account that hepatocytes have relatively low levels of cytosolic lipases. Elevated expression of p62 after CPE treatment further confirmed the effect of CPE on autophagy flux repair, since patients with known NAFLD demonstrate increased p62 accumulation upon liver biopsy (Gonzalez-Rodriguez et al. 2014).

The dose at which we detected a reduction in hepatic lipids and induction of autophagy *in vitro* was 50 μg/mL, which was similar to the plasma concentration evaluated from our previous animal study (Yao et al. 2015). Extrapolating from animals to humans, the approximate dose of 2 g CPE per 50 kg of body mass used in humans, roughly converted from that in mice (8 g/kg body mass), is reasonable and acceptable for daily consumption. Therefore, CP tea is suitable for daily use for treating NAFLD. CP leaf extract has been reported to contain many phytochemical constituents, including polysaccharides, triterpenoids, flavonoids, proteins, steroids, saponins, and phenolic compounds (Li et al. 2008; Ning et al. 2019). Thus, further studies are necessary to investigate the relationships between the active ingredients of CP and its mechanism of action.

## Conclusions

We have demonstrated that CPE has a potent effect in lowering the levels of hepatic lipids by activation of lipophagy in cell culture. Because there are no approved drug therapies for NAFLD (Lomonaco et al. 2013), understanding the mechanisms of the actions of natural dietary products such as CP offers further insight into developing drugs for the prevention and treatment of NAFLD. Our results may promote the utilization of CP as a healthy and functional beverage for preventing metabolic syndrome.
